# Phytoestrogen **α**-Zearalanol Attenuates Homocysteine-Induced Apoptosis in Human Umbilical Vein Endothelial Cells

**DOI:** 10.1155/2013/813450

**Published:** 2013-10-01

**Authors:** Teng Liu, Dan-dan Hou, Qian Zhao, Wei Liu, Pan-pan Zhen, Jian-ping Xu, Ke Wang, Hai-xia Huang, Xiao Li, Hui Zhang, Hai-bo Xu, Wen Wang

**Affiliations:** ^1^Department of Physiology and Pathophysiology, School of Basic Medical Sciences, Capital Medical University, You An Men Wai, Fengtai District, Beijing 100069, China; ^2^Department of Cell Biology, Municipal Laboratory for Liver Protection and Regulation of Regeneration, Capital Medical University, You An Men Wai, Fengtai District, Beijing 100069, China; ^3^Yanjing Medical College, Capital Medical University, 4 Dadong Road, Shunyi District, Beijing 101300, China

## Abstract

Hyperhomocysteinemia is an independent risk factor for cardiovascular diseases. The enhanced nitrative stress plays an important role in homocysteine-induced endothelial dysfunction. Previous studies have showed that phytoestrogen **α**-zearalanol alleviated endothelial injury in ovariectomized hyperhomocysteinemic rats; however, the underlying mechanism remains to be clarified. This study was to investigate the effects of **α**-zearalanol on homocysteine-induced endothelial apoptosis *in vitro* and explore the possible role of nitrative stress in these effects. Results showed that homocysteine (500 **μ**mol/L, 24 h) induced the apoptosis of human umbilical vein endothelial cells (HUVECs) obviously, and this effect was significantly attenuated by pretreatment with **α**-zearalanol (10^−8^~10^−6^ mol/L). Moreover, **α**-zearalanol downregulated proapoptotic protein Bax, upregulated antiapoptotic proteins Bcl-2 and Bcl-XL, and decreased the expression and activity of caspase-9. These findings demonstrated that **α**-zearalanol could effectively alleviate homocysteine-induced endothelial apoptosis, and this antiapoptosis effect might be related to the inhibition of the intrinsic pathway. Western blot indicated an enhanced 3-nitrotyrosine expression in HUVECs when challenged with homocysteine, which was attenuated by pretreatment with **α**-zearalanol. This result implied that inhibition of nitrative stress might play a role in the protective effect of **α**-zearalanol on endothelial cells. Such discovery may shed a novel light on the antiatherogenic activities of **α**-zearalanol in hyperhomocysteinemia.

## 1. Introduction

Hyperhomocysteinemia has been considered to be an independent risk factor for atherosclerosis, even a predictor of cardiovascular diseases [1, 2]. Endothelial dysfunction is an early event in the development of atherosclerosis and determines future vascular diseases' complications [3]. Hyperhomocysteinemia can induce apoptosis of endothelial cells directly [4], which accelerates endothelial injury. Production of superoxide, a potent reactive oxygen species (ROS), contributes significantly to endothelial dysfunction in hyperhomocysteinemia [5]. Similarly, disrupted nitric oxide (NO) signaling is a commonly reported outcome of hyperhomocysteinemia and a significant factor in cardiovascular diseases [6]. Nitrotyrosinylation of proteins occurs when superoxide quenches NO to form peroxynitrite (ONOO^−^), which binds tyrosine residues in proteins to produce 3-nitrotyrosine (3-NT). Widespread regulation of endothelial function is manifested through 3-NT modification of proteins involved in cell survival and matrix architecture [7]. Reports show that homocysteine increases inducible NO synthase (iNOS) expression and 3-NT formation in endothelial cells *in vitro*, and the enhanced nitrative stress is the major concern in homocysteine-induced endothelial dysfunction [8, 9].

Studies have showed that estrogen could reduce vascular endothelial injury induced by homocysteine [10]; nonetheless, the risk of developing breast and endometrial cancer in women taking estrogen has limited its clinical application [11–13]. Recently, studies have documented that plant derived phytoestrogens displayed cardiovascular protective effects similar to estrogen, meanwhile, without the potential negative effects that estrogen has [14]. Recently, *α*-zearalanol (*α*-ZAL), a plant-derived phytoestrogen, has been proposed as a potential replacement for estrogen [15]. *α*-ZAL is a reductive product of the fungus *Gibberella zeae* (*fusarium roseum* graminearum) metabolite zearalenone, which was isolated from culture medium of zearalenone and belongs to the *β*-resorcylate family [16]. In addition, *α*-ZAL is abundant in plants and vegetables including soybean, wheat, grape, radish, celery, spinach, and apple. Both *α*-ZAL and its parent compound zearalenone act as universal endogenous hormones for plant growth with *α*-ZAL being twice as effective as zearalenone, but less toxic. *α*-ZAL is rapidly metabolized in the body with few residues left in organs such as muscle, heart, liver, pancreas, kidney, and blood. Data from others and our group have revealed that *α*-ZAL had certain cardiovascular protective effects but less undesired effects [17–19]. Our previous studies have demonstrated that *α*-ZAL supplement could both improve the endothelium-dependent vascular relaxation function and alleviate endothelial morphological damage in ovariectomized hyperhomocysteinemie rats *in vivo*; this protective effect is similar to that of 17*β*-estradiol (17*β*-E_2_) [20]. However, the underlying mechanisms remain to be clarified. This study was designed to investigate the effects of *α*-ZAL on endothelial apoptosis induced by homocysteine in human umbilical vein endothelial cells (HUVECs) *in vitro* and explore the possible role of nitrative stress in this reaction.

## 2. Materials and Methods

### 2.1. Materials

*α*-ZAL, 17*β*-E_2_, DL-homocysteine, Type I collagenase, albumin from bovine serum (BSA), and 3-(4,5-dimethylthiazol-2-yl)-2,5-diphenyltetrazolium bromide (MTT) were obtained from Sigma-Aldrich (St. Louis, MO, USA). Low Serum Growth Supplement (LSGS) and 0.25% Trypsin-EDTA were obtained from Gibco-BRL (MD, USA). Fetal bovine serum (FBS) and RPMI-1640 medium were obtained from Hyclone (Logan, UT, USA). In situ Cell Death Detection Kit was purchased from Roche (Lewes, UK). AC-LEHD-AFC (caspase-9 activity assay substrate) was purchased from Enzo Life Sciences (Farmingdale, NY, USA). Rabbit anti-*β*-actin polyclonal antibody, rabbit anti-caspase-3 polyclonal antibody, rabbit anti-cleaved-caspase-3 monoclonal antibody (Asp175, 5A1E), and mouse anti-caspase-9 monoclonal antibody (C9) were procured from Cell Signaling Technology (Danvers, MA, USA). Anti-3-nitrotyrosine (MAB3248) was purchased from R&D Systems (Minneapolis, MN, USA). Rabbit anti-NOS3 (endothelial nitric oxide synthase, eNOS) (C-20) polyclonal antibody, goat anti-VE-cadherin (C-19) polyclonal antibody, mouse anti-Bcl-XL (H-5) monoclonal antibody, mouse anti-Bcl-2 (C-2) monoclonal antibody, mouse anti-Bax (B-9) monoclonal antibody, and HRP conjugated goat anti-rabbit IgG antibody, rabbit anti-mouse IgG antibody, and rabbit anti-goat IgG antibody were purchased from Santa Cruz Biotechnology (Santa Cruz, CA, USA).

### 2.2. Cell Culture and Identification

This investigation has been conducted according to the principles expressed in the Declaration of Helsinki. Umbilical cords for primary human umbilical vein endothelial cells (HUVECs) isolation were obtained from nine healthy births at Xuanwu Hospital Affiliated to Capital Medical University with written informed consent in accordance with the guidelines of research ethics committee of Capital Medical University and had been specifically approved by this committee. HUVECs were isolated from fresh human umbilical cords as previously described [19, 21]. HUVECs were cultured in RPMI-1640 medium containing 15% FBS, 2% LSGS, 100 IU/mL penicillin, and 10 *μ*g/mL streptomycin in a humidified incubator at 37°C with 5% CO_2_. The endothelial cells were identified by the presence of eNOS and VE-cadherin antigens and a typical “cobblestone” appearance. Endothelial cells of the third to fifth passages in the activel condition were used for experiments. Before different treatment, the medium was serum-starved with 1% FBS for 8 h first. If not stated otherwise, cells were preincubated with different concentrations of *α*-ZAL or 17*β*-E_2_ (10^−8^~10^−6^ mol/L), 30 min prior to the treatment with homocysteine (500 *μ*mol/L) for additional 24 h.

### 2.3. Cell Viability Assay

 Cells were seeded at a density of 1 × 10^5^ cells/well in 96-well plates for 12 h; then, the cells were cultured in low-serum (1% FBS) medium for another 8 h before drug treatment. Drug treatment groups were preincubated with different concentrations of *α*-ZAL or 17*β*-E_2_ (10^−9^~10^−6 ^mol/L), 30 min prior to the treatment with homocysteine (500 *μ*mol/L) for additional 24 h. The vehicle group changed medium at the same time without drug treatment. The cell viability was determined by MTT assay. Briefly, at the indicated time after different treatments, the culture supernatant was removed. The cells were washed with PBS and incubated with 10 *μ*L of 5 mg/mL MTT in culture medium at 37°C for 4 h. Then, culture medium with dye was removed, and 150 *μ*L of DMSO per well was added for formazan solubilization. The absorbance of converted dye was measured at a wavelength of 490 nm. The viability of cells was determined by the average values from triplicate readings, and presented as percentage of vehicle group.

### 2.4. TUNEL Immunofluorescence Staining Assay

Apoptosis was determined by morphological analysis following transferase-mediated dUTP nick-end labeling (TUNEL) staining. Briefly, cells grown on coverslips in 24-well plates after different treatments were fixed in 4% paraformaldehyde fixative for 30 min and then permeated with 0.1% Triton X-100 in 0.1% sodium citrate; cells were washed with PBS, 5 min × 3. Cells resuspended was in 50 *μ*L/well TUNEL reaction mixture (freshly prepared), lid added, and cells were incubated for 60 min at 37°C in the dark; then, cells were washed with PBS, 5 min × 3. Mounting medium with DAPI was used to fix the coverslips. Cells were then examined under a fluorescence microscope (Leica, Germany). The apoptotic cells showed green fluorescence. Tests were done in triplicate, counting more than 300 total cells per sample.

### 2.5. Western Blot Analysis

Cells were lysed in RIPA protein lysis buffer. Protein concentration was determined using BCA Protein Assay kit. A total of 50 *μ*g protein was loaded to 12% SDS-PAGE. Proteins were transferred to PVDF sequi-Blot membranes. Nonspecific binding sites were blocked for 2 h with 5% nonfat dried milk in TBS (20 mM Tris Base, 0.5 M NaCl, pH 7.5). The membranes were incubated overnight at 4°C with anti-caspase-3 (1 : 1000 dilution), anti-cleaved-caspase-3 (1 : 1000 dilution), anti-caspase-9 (1 : 1000 dilution), anti-Bax (1 : 200 dilution), anti-3-nitrotyrosine (3-NT, 1 : 800 dilution) or anti-*β*-actin (1 : 2000 dilution) antibodies. The membranes were then incubated with appropriate horseradish peroxidase-conjugated secondary antibodies and developed using an enhanced chemiluminescence detection system (ECL, Amersham, Poole, UK). Intensities of immunoreactive bands were measured using the Quantity One software and normalized to *β*-actin levels.

### 2.6. Immunohistochemistry Staining Assay

Cells grown on coverslips in 24-well plates after different treatments were First fixed with 4% paraformaldehyde fixative for 1 h then washed with PBS, 5 min × 3. Second, cells were incubated with 3% H_2_O_2_ in the dark for 10 min in order to block the endogenous peroxidase, and then, they were washed with PBS, 5 min × 3. Third, cells were incubated with 5% BSA for 20 min at room temperature to block the nonspecific binding of immunoglobulin and then Incubated with primary antibody (mouse anti-Bax, mouse anti-Bcl-2, or mouse anti-Bcl-XL) at 1 : 300 dilutions overnight at 4°C; then cells were washed with PBS, 5 min × 3. Fourth, cells were incubated with polymer auxiliary agent at 37°C for 15 min and then washed with PBS, 3 min × 2. Fifth, cells were incubated with horseradish peroxidase-anti-rabbit/anti-mouse IgG polymer at 37°C for 15 min and then washed with PBS, 3 min × 2. Sixth, cells were stained with DAB for 15 min at room temperature; then, cells were washed with ddH_2_O and dehydrated. Finally, cells were sealed with neutral gum and examined immediately with a light microscope (Leica, Germany). Five visual fields at different sites were taken in account for analysis per well cells. The intensities of immunocytochemistry staining were measured using the Image-Pro Plus 6.0 software.

The average of the five results from same well cells was considered to be one data for statistical analysis. The intensities of vehicle group were set as 100%, and intensities of other group were normalized to vehicle levels.

### 2.7. Caspase-9 Activity Assay

 The activity of caspase-9 was measured with chemiluminescence as we reported previously [22]. The substrate Ac-LEHD-pNA was used to determine caspase-9 activity according to the manufacturer's instructions. Briefly, cell tissues were homogenized in ice-cold lysis buffer and centrifuged at 12,000 rpm for 10 min at 4°C; 50 *μ*L of supernatant was then incubated with buffer containing 10 mM dithiothreitol and 5 *μ*L Ac-LEHD-pNA (the final concentration was 200 *μ*M) at 37°C for 1.5 h. Activity of caspase-9 was determined using a spectrophotometer at 405 nm (Molecular Devices, Sunnyvale, CA), and the results were expressed as -fold of the vehicle group.

### 2.8. Data Analysis

The results were expressed as mean ± S.D of at least three independent experiments. Statistical analysis was performed with SPSS 13.0. Statistical comparisons between two groups were performed using unpaired Student's *t*-test; comparisons between multiple groups were made by one-way ANOVA. Differences were considered statistically significant at a value of *P* < 0.05.

## 3. Results

### 3.1. Identification of Primary HUVECs

**Figure 1:**
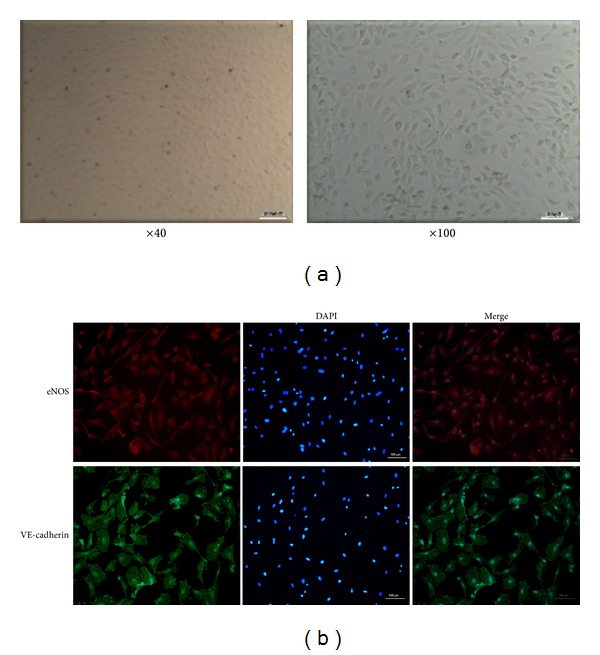
Characterization of primarily cultured human umbilical vein endothelial cells (HUVECs). (a) After 5 days of feeding, cells isolated from human umbilical cord vein showed a typical, cobblestone-like morphology of endothelial cells. (b) The markers of endothelial cells (eNOS and VE-cadherin) were positive with immunofluorescence staining (×100).

HUVECs grew as confluent monolayers with a cobblestone-like morphology (Figure 1(a)). The presence of markers of endothelial cells (eNOS and VE-cadherin) was confirmed by immunofluorescence staining (Figure 1(b)). 

### 3.2. Pretreatment with *α*-ZAL Improved Cell Viability of Homocysteine-Challenged HUVECs

**Figure 2:**
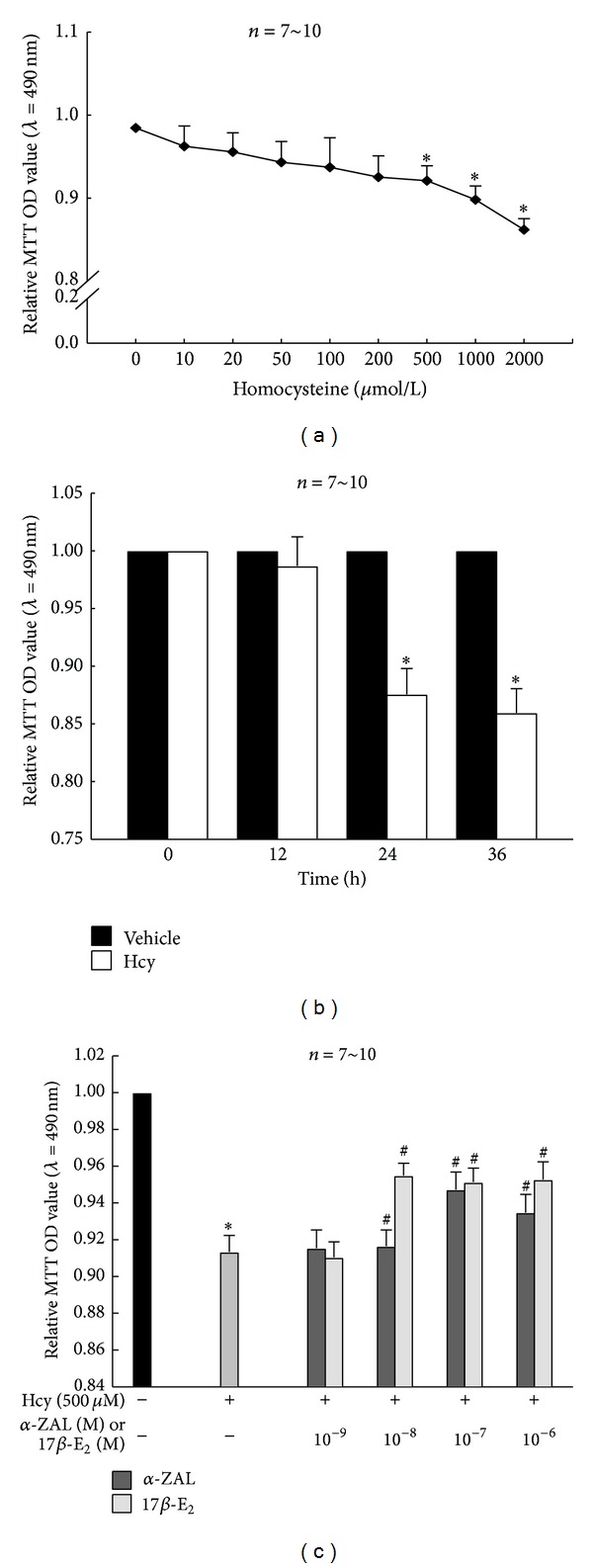
Pretreatment with *α*-ZAL improved the deceased cell viability induced by homocysteine with methyl thiazolyl-tetrazolium (MTT) assay in HUVECs. (a) Treatment with different concentrations of homocysteine on HUVECs for 24 h decreased cell viability in a dose-dependent manner, which became apparent at 500 *μ*mol/L. (b) Treatment with 500 *μ*mol/L homocysteine on HUVECs decreased cell viability in a time-dependent manner, which became apparent at 24 h. (c) Pretreatment with *α*-ZAL or 17*β*-E_2_ (10^−8^~10^−6^ mol/L) could significantly improve the decreased cell viability induced by homocysteine (500 *μ*mol/L, 24 h), which was similar to 17*β*-E_2_. Data were presented as mean ± SD. *P < 0.05 versus Vehicle, ^#^ *P* < 0.05 versus Hcy alone. *α*-ZAL: *α*-zearalanol; 17*β*-E_2_: 17*β*-estradiol; Hcy: homocysteine.

MTT assay showed that homocysteine decreased cell viability of HUVECs dose- and time-dependently. Treatment with different concentrations of homocysteine (0, 10, 20, 50, 100, 200, 500, 1000, and 2000 *μ*mol/L) on HUVECs for 24 h decreased cell viability in a dose-dependent manner, which became apparent at 500 *μ*mol/L (Figure 2(a)). Treatment with 500 *μ*mol/L homocysteine on HUVECs for different time intervals (12, 24, and 36 h) decreased cell viability in a time-dependent manner, which became apparent at 24 h (Figure 2(b)). Based on these results, 500 *μ*mol/L and 24 h were selected as the stimulating concentration and time interval of homocysteine in the later experiment.

Pretreatment with *α*-ZAL or 17*β*-E_2_ (10^−8^~10^−6^ mol/L) could significantly improve the decreased cell viability induced by homocysteine (500 *μ*mol/L, 24 h). Neither 10^−9^ mol/L *α*-ZAL nor 17*β*-E_2_ has a significant cell-protective effect in homocysteine-treated HUVECs (Figure 2(c)). This result indicated that 10^−8^~10^−6^ mol/L *α*-ZAL could exert protective effects on HUVECs and this protective effect was similar to that of 17*β*-E_2_. Based on these results, 10^−8^~10^−6^ mol/L were selected as the stimulating concentration of *α*-ZAL or 17*β*-E_2_ in the later experiment.

### 3.3. Pretreatment with *α*-ZAL Attenuated Apoptosis of Homocysteine-Challenged HUVECs

**Figure 3:**
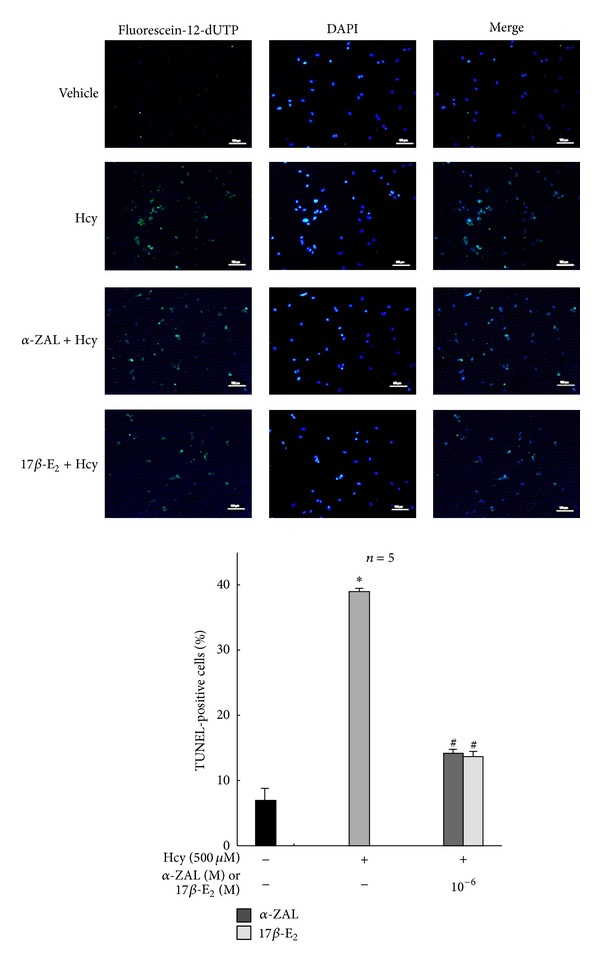
Pretreatment with *α*-ZAL attenuated apoptosis of homocysteine-challenged HUVECs—TUNEL fluorescence staining. The number of TUNEL-positive cells was significantly increased after treatment with 500 *μ*mol/L homocysteine for 24 h. Pretreatment with *α*-ZAL could attenuate the increased number of TUNEL-positive cells (only showing the picture of the highest concentration 10^−6^ mol/L). Data were presented as mean ± SD. *P < 0.05 versus Vehicle, ^#^ *P* < 0.05 versus Hcy alone. *α*-ZAL: *α*-zearalanol; 17*β*-E_2_: 17*β*-estradiol; Hcy: homocysteine.

**Figure 4:**
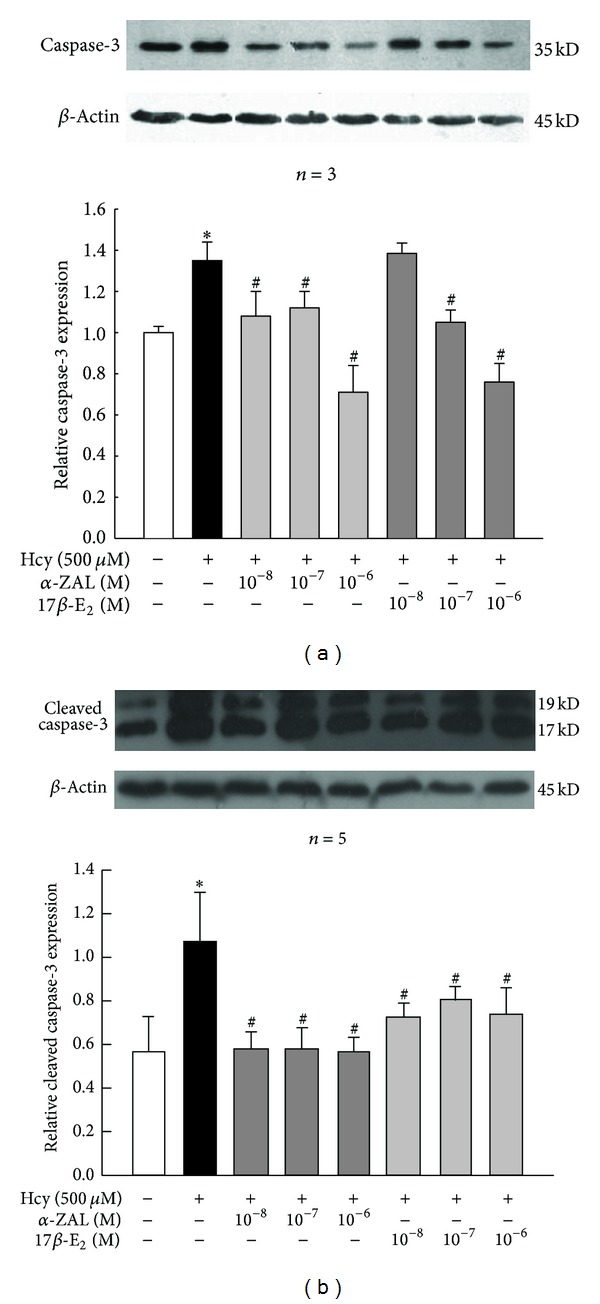
Pretreatment with *α*-ZAL attenuated apoptosis of homocysteine-challenged HUVECs—caspase-3/cleaved caspase-3 expression (Western blot). The expression of caspase-3 and cleaved caspase-3 was significantly increased after treatment with 500 *μ*mol/L homocysteine for 24 h. Pretreatment with *α*-ZAL could attenuate this effect. (a) Expression of caspase-3. (b) Expression of cleaved caspase-3. Data were presented as mean ± SD. *P < 0.05 versus Vehicle, ^#^ *P* < 0.05 versus Hcy alone. *α*-ZAL: *α*-zearalanol; 17*β*-E_2_: 17*β*-estradiol; Hcy: homocysteine.

Cell apoptosis was determined by TUNEL fluorescence staining and the expression of caspase-3 and cleaved caspase-3. Only minimal TUNEL-positive cells were observed in vehicle group, while the number of TUNEL-positive cells was found to be significantly increased after treatment with 500 *μ*mol/L homocysteine for 24 h (Figure 3). Both caspase-3 and cleaved caspase-3 protein levels were detected using Western Blot to confirm apoptosis. Cells treated with 500 *μ*mol/L homocysteine for 24 h showed more caspase-3 and cleaved caspase-3 expression than normal cells (Figure 4). All these indicated that homocysteine induced obvious apoptosis of HUVECs. 

Pretreatment with *α*-ZAL could attenuate HUVECs apoptosis induced by homocysteine. Both the number of TUNEL-positive cells and the expression of caspase-3/cleaved caspase-3 protein decreased after pretreatment with *α*-ZAL or 17*β*-E_2_. This protective effect of *α*-ZAL was similar to that of 17*β*-E_2_ (Figures 3 and 4).

### 3.4. Pretreatment with *α*-ZAL Reduced the Expression and Activity of Caspase-9 and the Expression of Proapoptotic Protein Bax and Enhanced the Expression of Prosurvival Protein Bcl-2 and Bcl-XL in Homocysteine-Challenged HUVECs

Apoptosis can be initiated through two pathways: the extrinsic pathway and the intrinsic pathway. The intrinsic pathway is mitochondrial-dependent, involving caspases (i.e., caspase-9, caspase-3, et al.) and Bcl-2 protein family (Bcl-2, Bax, Bcl-XL, et al.) [23]. The mitochondrial mechanism play an important role in endothelial cells apoptosis in hyperhomocysteinemia [24]. 

**Figure 5:**
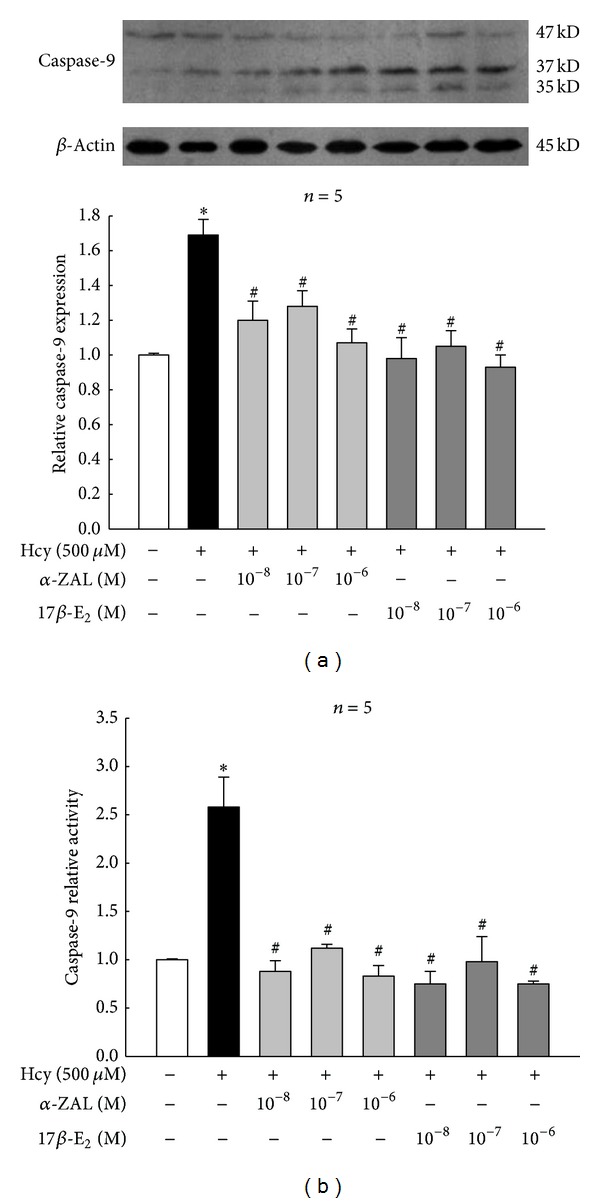
Pretreatment with *α*-ZAL reduced the expression and activity of caspase-9 in homocysteine-challenged HUVECs. The expression and activity of caspase-9 were significantly increased after treatment with 500 *μ*mol/L homocysteine for 24 h. Pretreatment with *α*-ZAL could attenuate this effect. (a) Expression of caspase-9 (Western blot). (b) Activity of caspase-9 (chemiluminescence). Data were presented as mean ± SD. *P < 0.05 versus Vehicle, ^#^ *P* < 0.05 versus Hcy alone. *α*-ZAL: *α*-zearalanol; 17*β*-E_2_: 17*β*-estradiol; Hcy: homocysteine.

**Figure 6:**
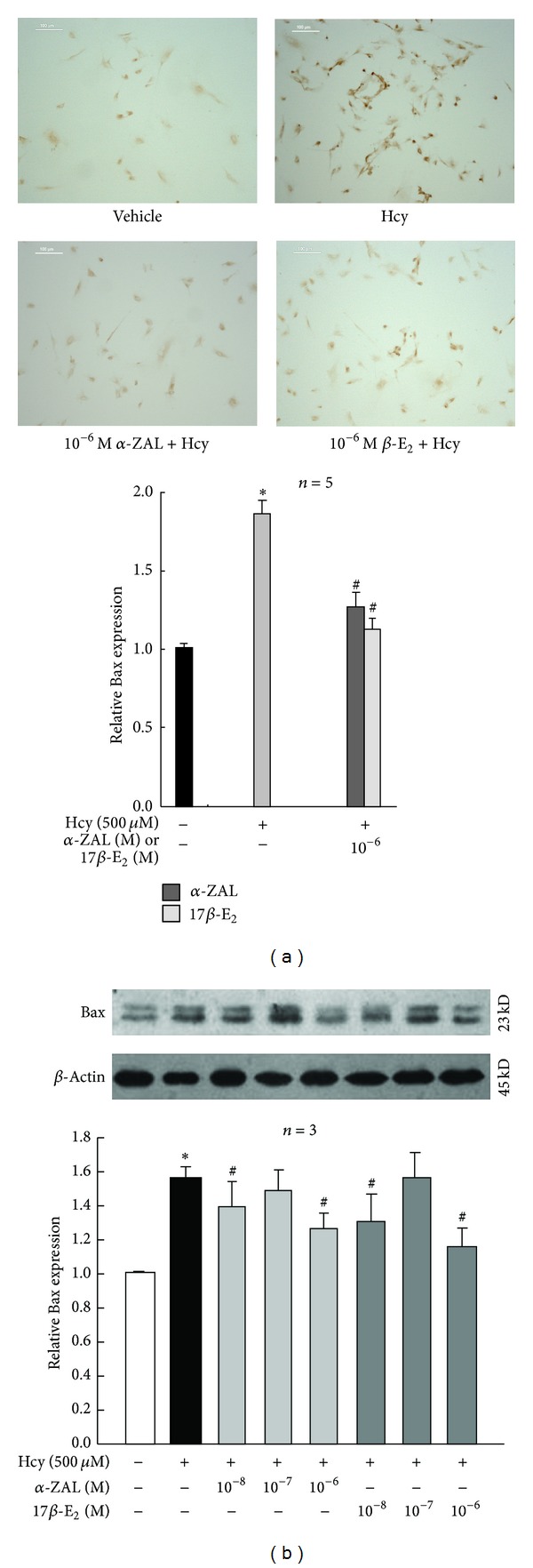
Pretreatment with *α*-ZAL reduced the expression of proapoptotic protein Bax in homocysteine-challenged HUVECs. The expression of proapoptotic protein Bax was significantly increased after treatment with 500 *μ*mol/L homocysteine for 24 h. Pretreatment with *α*-ZAL could reduce the expression of Bax. (a) Immunohistochemistry staining (only showing the picture of the highest concentration 10^−6^ mol/L). (b) Western blot. Data were presented as mean ± SD. *P < 0.05 versus Vehicle, ^#^ *P* < 0.05 versus Hcy alone. *α*-ZAL: *α*-zearalanol; 17*β*-E_2_: 17*β*-estradiol; Hcy: homocysteine.

**Figure 7:**
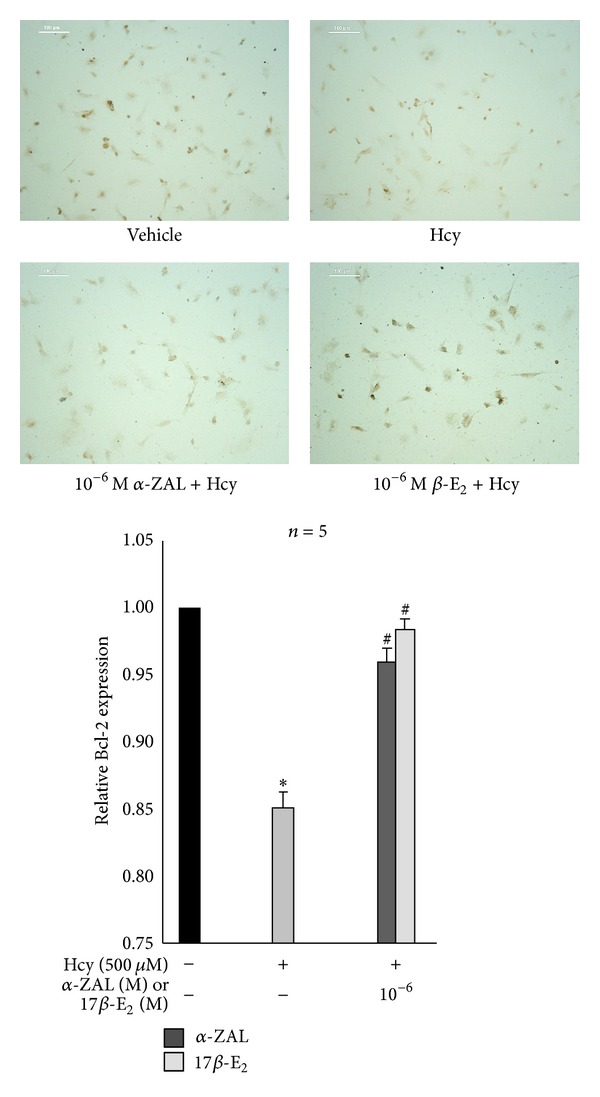
Pretreatment with *α*-ZAL enhanced the expression of prosurvival protein Bcl-2 in homocysteine-challenged HUVECs (immunohistochemistry staining, only showing the picture of the highest concentration 10^−6^ mol/L). The expression of prosurvival protein Bcl-2 was significantly decreased after treatment with 500 *μ*mol/L homocysteine for 24 h. Pretreatment with *α*-ZAL could enhance the expression of Bcl-2. Data were presented as mean ± SD. *P < 0.05 versus Vehicle, ^#^ *P* < 0.05 versus Hcy alone. *α*-ZAL: *α*-zearalanol; 17*β*-E_2_: 17*β*-estradiol; Hcy: homocysteine.

**Figure 8:**
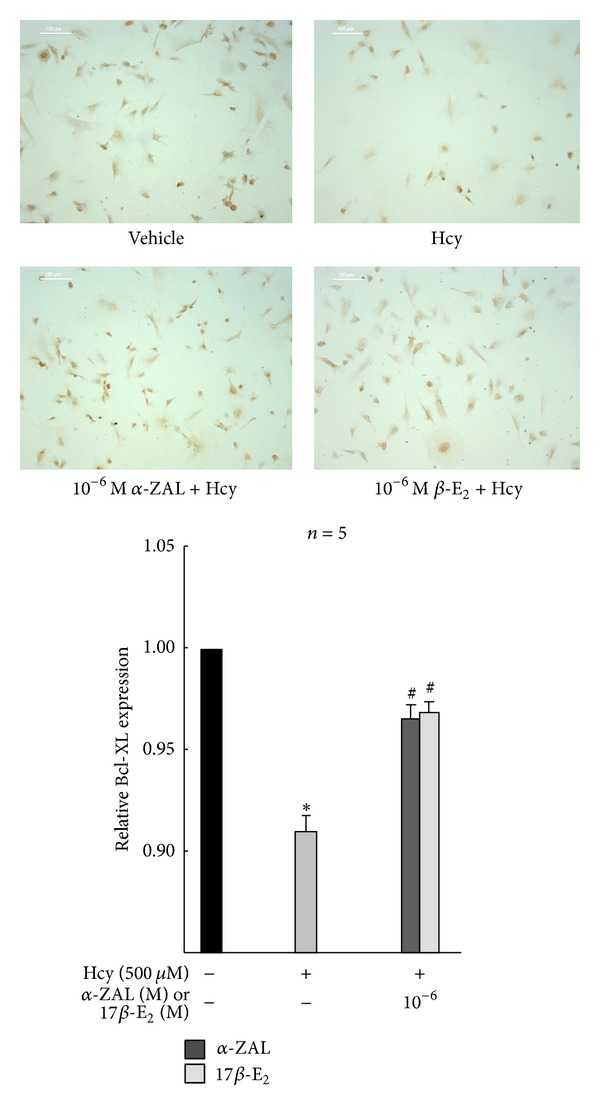
Pretreatment with *α*-ZAL enhanced the expression of prosurvival protein Bcl-XL in homocysteine-challenged HUVECs (immunohistochemistry staining, only showing the picture of the highest concentration 10^−6^ mol/L). The expression of prosurvival protein Bcl-XL was significantly decreased after treatment with 500 *μ*mol/L homocysteine for 24 h. Pretreatment with *α*-ZAL could enhance the expression of Bcl-XL. Data were presented as mean ± SD. *P < 0.05 versus Vehicle, ^#^ *P* < 0.05 versus Hcy alone. *α*-ZAL: *α*-zearalanol; 17*β*-E_2_: 17*β*-estradiol; Hcy: homocysteine.

Western blot, immunohistochemistry staining, and chemiluminescence indicated that homocysteine could increase the expression and activity of caspase-9 (Figure 5), upregulate the expression of proapoptotic protein Bax (Figure 6), and downregulate the expression of prosurvival proteins Bcl-2 (Figure 7) and Bcl-XL (Figure 8) in HUVECs, indicating that the activation the mitochondrial pathway in the homocysteine-induced endothelial cells apoptosis, which was accordant with the result of Tyagi et al. [24]. Furthermore, the effect of *α*-ZAL on mitochondrial pathway-related proteins was assayed. Similar to 17*β*-E_2_, pretreatment with *α*-ZAL could reverse these above changes, indicating that the antiapoptosis effect of *α*-ZAL on HUVECs may be related to inhibition of the intrinsic pathway of apoptosis.

### 3.5. Pretreatment with *α*-ZAL Reduced the Expression of 3-Nitrotyrosine in Homocysteine-Challenged HUVECs

**Figure 9:**
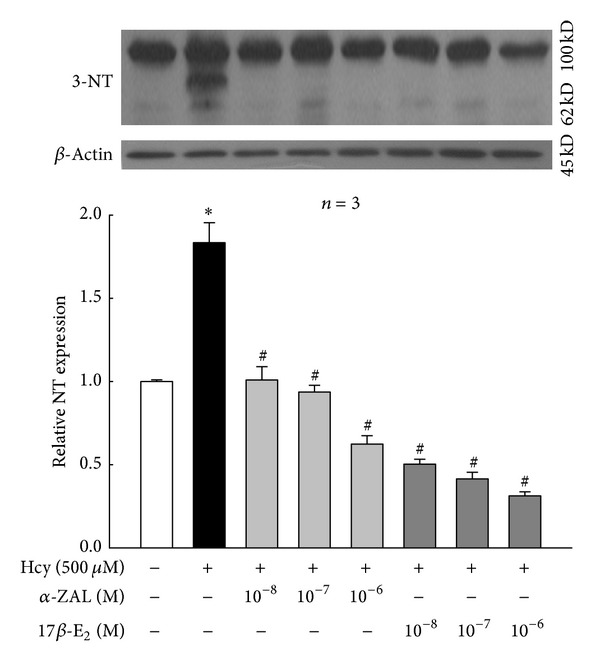
Pretreatment with *α*-ZAL reduced the expression of 3-nitrotyrosine in homocysteine-challenged HUVECs (Western blot). The expression of 3-nitrotyrosine was significantly increased after treatment with 500 *μ*mol/L homocysteine for 24 h. Pretreatment with *α*-ZAL could attenuate this effect. Data were presented as mean ± SD. *P < 0.05 versus Vehicle, ^#^ *P* < 0.05 versus Hcy alone. *α*-ZAL: *α*-zearalanol; 17*β*-E_2_: 17*β*-estradiol; Hcy: homocysteine; 3-NT: 3-nitrotyrosine.

The generation of peroxynitrite was monitored by the formation of 3-nitrotyrosine (3-NT) [25]. Western blot indicated the enhanced 3-NT expression in HUVECs challenged with homocysteine, which was attenuated by pretreatment with *α*-ZAL (Figure 9). This result implied that inhibition of nitrative stress might play a role in the protective effect of *α*-ZAL on homocysteine-induced apoptosis in endothelial cells. 

## 4. Discussion

Two novel contributions were made in this investigation. First, phytoestrogen *α*-ZAL could effectively alleviate homocysteine-induced HUVECs apoptosis; the antiapoptosis effect of *α*-ZAL on HUVECs may be related to inhibition of the intrinsic pathway. Second, pretreatment with *α*-ZAL could attenuate the expression of 3-nitrotyrosine in homocysteine-treated HUVECs, which implied that inhibition of nitrative stress might contribute to the protective effect of *α*-ZAL on homocysteine induced apoptosis in endothelial cells. 

Hyperhomocysteinemia is an established risk factor for atherosclerosis. Endothelial apoptosis representes the critical event for the initiation of atherosclerosis [26]. Hyperhomocysteinemic can induce apoptosis of endothelial cells directly [4], which accelerated endothelial injury. Our previous studies demonstrated that the ovariectomized hyperhomocysteinemia rats have developed obvious vascular endothelial damage; *α*-ZAL, a native phytoestrogen, could alleviate the impairment of homocysteine on vascular endothelium *in vivo*; this protective effect was similar to that of 17*β*-estradiol (17*β*-E_2_) [20]. However, the mechanisms by which *α*-ZAL attenuate endothelial dysfunction remain to be clarified. Based on the previous work, in this study, we explored the effects of *α*-ZAL on homocysteine-induced endothelial apoptosis in primarily cultured HUVECs *in vitro*. Our results showed that pretreatment with *α*-ZAL could effectively alleviate homocysteine-induced endothelial apoptosis; this protective effect was somewhat similar to that of 17*β*-E_2_. Moreover, the anti-apoptosis effect of *α*-ZAL on HUVECs might be related to the inhibition of the intrinsic pathway.

Generally, apoptosis can be initiated through two pathways: the extrinsic pathway and the intrinsic pathway. The extrinsic pathway is activated by extracellular signals that interact with cell surface receptors (surface receptors mediated), involving caspase-8, caspase-7, caspase-6, and caspase-3. The intrinsic pathway is mitochondrial-dependent, involving caspases (i.e., caspase-9, caspase-7, caspase-6, and caspase-3) and Bcl-2 protein family (Bcl-2, Bax, Bcl-XL, et al.) [23]. Mitochondria played an important role in the regulation of the process of apoptosis as the center of energy production and metabolic center of eukaryotes. Among the various caspases, caspase-9 and caspase-3 appear to be very crucial in cell apoptosis. The release of cytochrome C in mitochondria caused by apoptotic signals could activate caspase-9 and caspase-3, leading to cell apoptosis. The proapoptosis proteins Bax could promote cytochrome C release through opening of permeability transition pore (PTP); meanwhile, the prosurvival proteins Bcl-2 and Bcl-XL could prevent the opening of PTP through competition or directly inhibiting PTP opening, by which they have anti-apoptotic effect. The mitochondrial mechanism plays an important role in endothelial cells apoptosis in hyperhomocysteinemia. Tyagi et al. [24] reported that homocysteine-mediated endothelial cell apoptosis occurs in part by disturbing PTP, which results in subsequent release of cytochrome C and activation of caspase-9 and caspase-3, leading to cell apoptosis. In this study, we found that pretreatment with *α*-ZAL reduced the expression of proapoptotic protein Bax, improved the expression of prosurvival proteins Bcl-2 and Bcl-XL, and reduced the expression and activity of caspase-9 of HUVECs challenged with homocysteine, indicating that the anti-apoptosis effect of *α*-ZAL on HUVECs maybe through the inhibition of the intrinsic pathways, which was similar to that of 17*β*-E_2_. 

Recently, the role of nitrative stress in homocysteine-induced endothelial dysfunction had drawn much attention. As we know, high levels of homocysteine may promote oxidative stress in endothelial cells as a result of production of reactive oxygen species [27], which has been strongly implicated in the development of atherosclerosis. Production of superoxide, a potent reactive oxygen species (ROS), contributes significantly to endothelial dysfunction in hyperhomocysteinemia [5]. Meanwhile, disrupted nitric oxide (NO) signaling is a commonly reported outcome of hyperhomocysteinemia and a significant player in cardiovascular diseases [6]. Nitrotyrosinylation of proteins occurs when superoxide quenches NO to form peroxynitrite (ONOO^−^), which binds tyrosine residues in proteins to produce 3-nitrotyrosine (3-NT). The generation of peroxynitrite was monitored by the formation of 3-NT. Peroxynitrite is a highly reactive oxidant that mediates a variety of biological processes including inhibition of key metabolic enzymes, lipid peroxidation, nitration of the protein tyrosine residues, and reduction of cellular antioxidant defenses by oxidation of thiol pools [28]. Widespread regulation of endothelial function is manifested through 3-NT modification of proteins involved in cell survival and matrix architecture [7]. There are reports that homocysteine increases inducible NO synthase (iNOS) expression and 3-NT formation in endothelial cells *in vitro*, and the enhanced nitrative stress plays an important role in homocysteine-induced endothelial dysfunction [8, 9]. Duan et al. [29] had reported that *α*-ZAL may antagonize homocysteine-induced ROS accumulation, which may contribute to *α*-ZAL-induced beneficial effect on endothelial function. Based on these pieces of work, we further explored whether *α*-ZAL might inhibit the nitrative stress in homocysteine-treated HUVECs or not. 3-NT, as the footprint of peroxynitrite, was an indicator of nitrative stress. Our results indicated the enhanced 3-NT expression in HUVECs challenged with homocysteine, which was attenuated by pretreatment with *α*-ZAL. This result implied that it was possible that inhibiting of nitrative stress might play a role in the protective effect of *α*-ZAL on homocysteine-induced apoptosis in endothelial cells. By far the accurate mechanisms involved in *α*-ZAL's inhibition of nitrative stress in endothelium still remain to be clarified, which is also the limitation of this study. El-Remessy et al. [30] have reported that peroxynitrite caused tyrosine nitration of PI3-kinase, inhibiting the activity of Akt-1 kinase, and increasing the activity of p38 MAP kinase, resulting in inactivated VEGF survival signaling and at last accelerated endothelial cell apoptosis. Would this pathway be possibly involved in the action of *α*-zearalanol? Additional time and effort are required to clarify the underlying mechanisms. 

In conclusion, this study demonstrated that phytoestrogen *α*-ZAL could effectively alleviate homocysteine-induced HUVECs apoptosis; the anti-apoptosis effect of *α*-ZAL on HUVECs may be related to the inhibition of the intrinsic pathway. Moreover, inhibition of nitrative stress might play a role in the protective effect of *α*-ZAL on homocysteine-induced HUVECs apoptosis. Although the underlying mechanisms responsible for this effect need to be elucidated, this study may shed a novel light on the antiatherogenic activities of *α*-ZAL in hyperhomocysteinemia.
